# Radiographic outcomes of a standardized versus conventional reduction technique in proximal humerus fractures: a matched cohort study

**DOI:** 10.1016/j.jseint.2026.101750

**Published:** 2026-06-18

**Authors:** Christiane Barthel, Tim Schneller, Daniela Brune, Frederik Bellmann, Asimina Lazaridou, Markus Scheibel

**Affiliations:** a Department of Shoulder and Elbow Surgery, Schulthess Klinik, Zurich, Switzerland; b Teaching, Research and Development, Schulthess Klinik, Zurich, Switzerland; c Department of Anesthesiology, Brigham and Women's Hospital, Harvard Medical School, Boston, MA, USA; d Center for Musculoskeletal Surgery, Charité-Universitaetsmedizin Berlin, Berlin, Germany

**Keywords:** Proximal humerus fracture, Open reduction and internal fixation, Standardized surgical technique, Reduction quality, Medial calcar, Neck–shaft angle, Locking plate fixation

## Abstract

**Background:**

Displaced proximal humerus fractures are common in the elderly and often challenging to reduce anatomically, particularly in comminuted or osteoporotic bone. Variability in surgical approaches and fixation techniques contributes to inconsistent reduction quality and clinical outcomes. A standardized step-by-step reduction technique using temporary transhumeral K-wire retention has been proposed to improve reproducibility, but comparative evidence remains limited. The aim of this study is to compare radiographic outcomes between a standardized stepwise reduction technique and a non-standardized method in proximal humerus fractures and to determine whether the standardized approach is associated with improved anatomical reduction and medial calcar reconstruction.

**Methods:**

In this single-center, retrospective matched-pair study, 72 patients with proximal humerus fractures treated with open reduction and internal plate fixation between January 2018 and June 2025 were analyzed. Thirty-six patients underwent the standardized reduction and temporary transhumeral K-wire retention technique (Group 1) and 36 underwent the conventional (nonstandardized) technique (Group 2). Patients were matched by fracture type using the Mayo Clinic–Fundación Jiménez Díaz classification and sex. The primary end point was assessed using a 3-item composite checklist (head–shaft displacement, head–shaft alignment, and cranialization of the greater tuberosity), along with separate evaluation of medial support. Associations between surgical technique and outcomes were assessed using ordinal logistic regression for surgical reduction score; logistic regression for medial calcar reconstruction; and linear regression for change in neck–shaft angle (NSA), adjusted for age.

**Results:**

Group 1 was associated with significantly higher odds of achieving a superior reduction score compared with Group 2 (odds ratio = 11.88, 95% confidence interval: 3.97–97.0, *P* < .001), indicating a substantially increased likelihood of anatomic alignment. Medial calcar reconstruction was significantly more frequent in Group 1 (odds ratio = 4.85, 95% confidence interval: 1.41–16.89, *P* = .013). No significant difference in the change in NSA was observed between techniques (*P* = .56), with both groups demonstrating similar mean pre-operative and post-operative NSA.

**Conclusion:**

The standardized reduction and retention technique was associated with higher radiographic reduction scores, more frequent medial calcar reconstruction, and more consistent post-operative alignment. These findings suggest improved radiographic reproducibility, particularly in complex or osteoporotic fractures. Prospective multicenter studies are needed to determine whether these radiographic differences translate into improved clinical and functional outcomes.

Proximal humerus fractures account for approximately 4–5% of all fractures and represent the third most common fracture in the elderly, reflecting their increasing relevance in an aging population.^4^^,^^13^^,^^19^ Their complex morphology and the common presence of osteoporotic bone frequently make anatomic reduction challenging,^3^^,^^4^ even for experienced surgeons. Consequently, many surgeons rely on individual intraoperative strategies or personal “styles,” which introduce variability in reduction quality and complicate both reproducibility and surgical training.

Locking plate fixation is the predominant treatment for displaced and complex fracture types,^13^^,^^23^ yet complication rates remain considerable. Varus malunion, screw perforation, and avascular necrosis are among the most frequently reported problems with controlled fragment handling,^1^^,^^3^ and post-operative loss of reduction, reported in 15–30% of patients, substantially increases reoperation rates to up to 40%.^8^^,^^14^ Achieving and maintaining anatomic alignment is therefore central to successful fixation. Key parameters include restoration of the head–shaft angle, correct tuberosity position, and reliable medial calcar support, all of which are strongly associated with fracture stability, risk of varus collapse, and functional outcome. Consequently, malreduction of crucial fragments correlates with higher failure and revision rates.^6^^,^^14^^,^^16^^,^^17^^,^^21^^,^^22^^,^^25^^,^^28^

To address this issue, Minkus and Scheibel^15^ introduced a standardized, stepwise technique for reduction and fixation of proximal humeral fractures. The standardized reduction and retention technique used in this study is based on this approach. Central to this protocol is reproducible restoration of the medial calcar which is intended to enhance stability and retention. Kwisda et al^12^ demonstrated encouraging clinical and radiological results using this approach, including low rates of primary, technique-related complications. Nevertheless, a significant gap persists: the standardized method has not been directly evaluated against the individualized surgical techniques in a matched comparative study.

Thus, evidence is lacking on whether standardization truly improves radiographic reduction quality and consistency beyond surgeon-dependent nonstandardized approaches.

The aim of this study was to compare radiographic results between the standardized and nonstandardized reduction, retention, and fixation technique. By evaluating key alignment parameters before and after surgery, we assessed whether this standardized technique was associated with improved reproducibility and quality of anatomic reconstruction.

## Materials and methods

**Figure 1:**
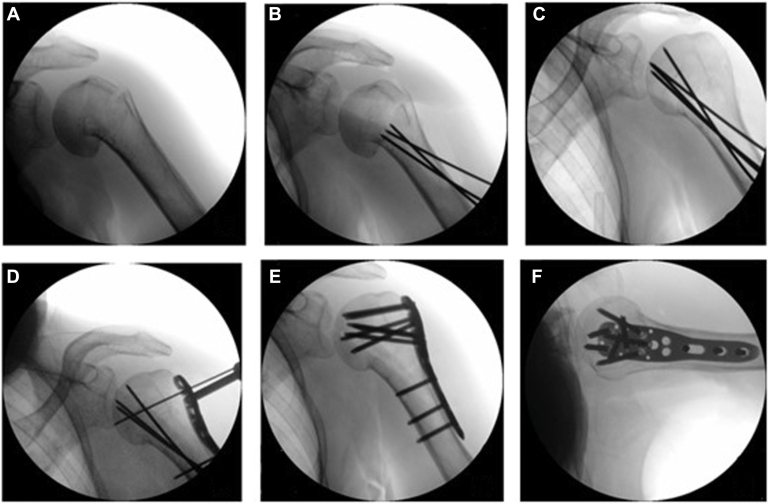
Intraoperative images illustrating the standardized reduction and retention technique. (**A**) Pre-operative image-intensifier evaluation. (**B**) Percutaneous K-wire insertion into the humeral shaft to the fracture line. (**C**) Anatomic head reduction and K-wire advancement into subchondral bone. (**D**) Plate introduction and temporary fixation with K-wires. (**E** and **F**) Final construct and post-operative image-intensifier evaluation.

**Figure 2:**
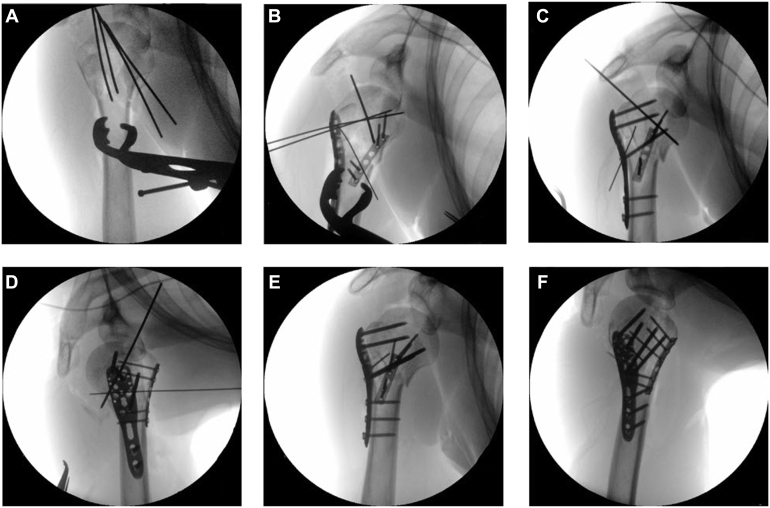
Intraoperative images illustrating an individualized surgical technique. (**A**) Manual fracture reduction and temporary fixation with K-wires inserted from caudal and cranial directions. (**B**) Additional fixation of a calcar fragment using a one-third tubular plate and temporary fixation of the locking plate with K-wires. (**C** and **D**) Definitive angular stable plate fixation with sequential repositioning of K-wires to prevent reduction loss. (**E** and **F**) Final construct and post-operative image-intensifier evaluation.

This single-center retrospective study was approved by the local ethics committee (BASEC-Nr. 2025-01620) and conducted at our institution. All patients provided general consent, and the study was performed in compliance with ethical standards for retrospective analyses. The standardized reduction and retention technique was compared with conventional individualized strategies employed by experienced upper extremity surgeons. Patients treated between January 2018 and June 2025 for proximal humerus fractures with open reduction and internal plate fixation were included. All patients were followed post-operatively with plain radiographs. Exclusion criteria were conservative treatment, alternative fixation methods (eg, intramedullary nailing), or fracture types limited to isolated greater tuberosity, head-split, impression, or shaft fractures. Patients were assigned to either the standardized group, Group 1 — treated using the structured protocol (Fig. 1), or Group 2 — treated using an individualized surgical technique (Fig. 2). Conventional (nonstandardized) techniques were defined as surgeon-dependent approaches without a universally predefined stepwise protocol. Individual surgeons may follow their own consistent routines; however, no common standardized sequence is applied, and approaches differ with regard to reduction sequence, fragment handling, temporary fixation, medial calcar reconstruction, and plate positioning.

The standardized reduction and retention technique is performed as follows: A standard deltopectoral approach is used. The rotator cuff tendons are armed with no. 5 nonabsorbable suture at the tendinous-osseous junction to primarily assist with anatomic reduction and secondarily neutralize the pull effects of the rotator cuff. Three percutaneous 2.0 mm K-wires are inserted retrograde into the humeral shaft toward the fracture line (Fig. 1*B*). The humeral head fragment is reduced anatomically, with emphasis on medial buttress integrity, and the K-wires are advanced into the subchondral bone for stabilization (Fig. 1*C*). The tuberosity fragments are then reduced. Following anatomic reduction, an angular stable locking plate is introduced and temporarily fixed with 1.6 mm K-wires (Fig. 1*D*). After fluoroscopic evaluation of plate position, the plate is secured with a 3.5 mm cortical screw before locking screws are inserted. The K-wires are removed, the nonabsorbable sutures are tightened, and the final construct is reevaluated for plate impingement and stability in various shoulder positions (Fig. 1, *E* and *F*).

In contrast, individualized surgical techniques often start with manual reduction of the fracture fragments, guided by anatomic landmarks. Once the fragments are aligned, fixation is achieved with K-wires, typically placed from various directions (Fig. 2). This approach relies on the surgeon's ability to maintain the reduction while securing the fragments using K-wires before proceeding to final fixation. Nonabsorbable sutures were placed through the rotator cuff tendons and secured to the plate to assist with reduction and maintain stable fixation of the tuberosities.

Reduction quality was assessed using a score ranging from 0 to 3 based on post-operative radiographic parameters as demonstrated by Schnetzke et al^25^ (head-shaft displacement of >5 mm, head-shaft alignment, and cranialization of the greater tuberosity of >5 mm). Secondary outcomes included the analysis of medial calcar reconstruction and the neck–shaft angle (NSA) only.

All imaging data, including standard radiographs and computed tomography (CT) scans, were retrieved from the clinical information system. Post-operative imaging consisted of standard radiographs (and CT scans when available), obtained approximately 6 weeks after surgery. All radiographic measurements were performed by a single experienced assessor. To assess interobserver reliability, all cases were independently reevaluated by a second fellowship-trained shoulder surgeon using the same radiographic parameters, including the surgical reduction score (SRS) and pre-operative and post-operative neck–shaft angle measurements. Treatment allocation was not randomized and was primarily based on surgeon preference and familiarity with the standardized technique. Each technique was performed by 4 different surgeons. Data extraction was performed by a single reviewer using predefined criteria. The standardized technique was defined by the use of a structured stepwise reduction protocol with temporary K-wire retention. In contrast, the conventional (nonstandardized) group included cases without this predefined protocol, with variability in reduction sequence, fragment handling, and use (or absence) of temporary fixation and adjunct techniques such as sutures.

After identifying patients in Group 1, a 1:1 matched control group was selected. Matching was performed based on fracture type, using the Mayo Clinic–Fundación Jiménez Díaz classification, and sex. In total, 72 patients (36 per group) were included. Data were processed using research electronic data capture. Statistical analyses were performed using R Core Team (R Foundation for Statistical Computing, Vienna, Austria.)^7^ Continuous variables were tested for normality (Shapiro-Wilk test). Standardized mean differences were calculated to assess balance after matching. Ordinal logistic (proportional-odds) and linear regression models were used to evaluate the association between surgical technique and outcomes. Models were adjusted for age, as propensity score matching did not achieve complete balance across groups. Statistical significance was set at *P* < .05.

## Results

A total of 72 patients were included in the final analysis, 36 in Group 1 and 36 matched controls in Group 2. The groups were well balanced in terms of age (55.7 ± 12.2 vs. 51.9 ± 15.0 years, *P* = .23), sex distribution (69% female in both groups), and American Society of Anesthesiologists classification (*P* = .98). No significant differences were observed in surgery duration (143.2 ± 33.1 vs. 132.4 ± 63.0 min, *P* = .37) or body mass index (25.7 ± 6.6 vs. 25.5 ± 5.1 kg/m^2^, *P* = .92). Fracture type distribution according to the Mayo Clinic–Fundación Jiménez Díaz classification and pre-operative neck–shaft angle (144.9 ± 23.7° vs. 145.0 ± 24.9°, *P* = .99) were comparable between groups, confirming appropriate matching and baseline equivalence prior to outcome analyses. Mean follow-up was 12.8 ± 6.6 months in Group 1 and 17.6 ± 15.9 months in Group 2 (*P* = .096). Complications requiring revision surgery occurred in 2 patients (5.6%) in Group 1 and 8 patients (22.2%) in Group 2 (*P* = .041). In Group 1, revision surgery was required in 2 cases of avascular necrosis. In Group 2, revision procedures were performed for avascular necrosis (n = 4), loss of reduction (n = 2), hardware loosening (n = 1), and malreduction (n = 1) (Table I).

**Table I:** Baseline characteristics of group 1 and group 2.

Variable	Group 2	Group 1	*P* value
N	36	36	
Age (mean ± SD)	55.7 ± 12.2	51.9 ± 15.0	.23
Sex (n, %)			–
Female	25 (69)	25 (69)	
Male	11 (31)	11 (31)	
ASA risk classification (n, %)			.98
1	9 (26)	10 (29)	
2	22 (65)	22 (63)	
3	3 (9)	3 (9)	
Surgery duration (min)	143.2 ± 33.1	132.4 ± 63.0	.37
BMI (mean ± SD)	25.7 ± 6.6	25.5 ± 5.1	.92
Mayo FJD classification (n, %)			–
SN	2 (6)	2 (6)	
SN-GT	2 (6)	2 (6)	
SN-GT-LT	6 (17)	6 (17)	
VL-GT	2 (6)	2 (6)	
VL-GT-LT	13 (36)	13 (36)	
VPM	2 (6)	2 (6)	
VPM-GT	5 (14)	5 (14)	
VPM-GT-LT	4 (11)	4 (11)	
Neck–shaft angle PreOP (mean ± SD)	144.9 ± 23.7	145.0 ± 24.9	.99
Follow-up time (in month (mean ± SD))	17.6 ± 15.9	12.8 ± 6.6	.096
Complications requiring revision surgery (n/%)	8 (22.2%)	2 (5.6%)	.041

*BMI*, body mass index; *PreOP*, preoperative; *SD*, standard deviation*; ASA,* American Society of Anesthesiologists; *FJD*, Mayo Clinic–Fundación Jiménez Díaz; *SN*, surgical neck; *SN-GT*, surgical neck + greater tuberosity; *SN-GT-LT*, surgical neck + greater tuberosity + lesser tuberosity; *VL-GT*, valgus-impacted + greater tuberosity; *VL-GT-LT*, valgus-impacted + greater tuberosity + lesser tuberosity; *VPM*, varus posteromedial; *VPM-GT*, varus posteromedial + greater tuberosity; *VPM-GT-LT*, varus posteromedial + greater tuberosity + lesser tuberosity.

### Quality of reduction

Quality of reduction, assessed using the SRS, was markedly superior in Group 1 compared with Group 2. Anatomic reduction (SRS = 3) was achieved in 35 of 36 fractures (97%) in Group 1 versus 17 of 36 (47%) in Group 2. Poor or terrible reductions (SRS ≤1) occurred exclusively in Group 2. Age-adjusted proportional odds ordinal logistic regression showed that Group 1 was associated with significantly higher odds of achieving a superior SRS (odds ratio = 11.88, 95% confidence interval [CI]: 3.97–97.0, *P* < .001), indicating a substantially increased likelihood of achieving anatomic alignment relative to Group 2.

## Medial calcar reconstruction

Reconstruction of the medial calcar was achieved in 31 patients (86%) in Group 1, compared with 14 patients (39%) in Group 2. Age-adjusted logistic regression analysis demonstrated that the standardized technique was associated with significantly higher odds of medial calcar reconstruction (odds ratio = 4.9, 95% CI: 1.41–16.89, *P* = .013).

### Neck–shaft angle

The mean pre-operative NSA was similar between groups (Group 1 145.0° ± 24.9° vs. Group 2 144.9° ± 23.7°). Post-operatively, the mean NSA was 139.0° ± 13.7° in Group 2 and 137.3° ± 6.3° in Group 1, corresponding to a mean change in NSA of −5.9° ± 22.5° and −7.7° ± 23.2°, respectively. Age-adjusted linear regression analysis showed no significant difference in NSA change between techniques (β = −3.15, 95% CI: −13.79 to 7.49, *P* = .556; Table II).

**Table II:** Regression models evaluating the association between surgical technique and radiographic outcomes.

Outcome/model	Effect estimate	95% CI	*P* value
Model 1: proportional-odds logistic regression Superior reduction (SRS) Model 2: logistic regression	OR = 11.88	3.97–97.0	<.001
Medial calcar reconstruction Model 3: linear regression	OR = 4.90	1.41–16.89	.013
Change in neck–shaft angle (ΔNSA)	β = −3.15	−13.79 to 7.49	.556

*SRS*, surgical reduction score; *ΔNSA*, change in neck–shaft angle; *OR*, odds ratio; *CI*, confidence interval.

All models adjusted for age.

## Interobserver reliability

Interobserver reliability between the 2 assessors demonstrated good to substantial agreement across all radiographic measures, with weighted kappa of 0.64 and 0.66 for the SRS and intraclass correlation coefficient (3,1) of 0.82 (0.72–0.88) and 0.72 (0.58–0.81) for pre-operative and post-operative NSA, respectively.

## Discussion

Our findings show that the standardized reduction, retention, and fixation technique is associated with improved quality and consistency of anatomic reconstruction in proximal humerus fractures. The standardized technique was associated with more reliable restoration of medial calcar support and head–shaft alignment, key factors for maintaining mechanical stability and avoiding secondary displacement.^1^^,^^3^ This is clinically important because loss of reduction remains one of the most common and consequential complications after locking plate fixation, occurring in up to one-third of cases^2^^,^^10^ and contributing to overall complication rates approaching 49% in some series.^9^^,^^26^ Once displacement occurs, reoperation rates rise substantially, reaching 20–40% in affected patients.^8^^,^^14^ As the quality of the initial reduction strongly correlates with functional outcomes and revision risk,^14^^,^^20^ a technique associated with more reproducible alignment and higher rates of medial support in our cohort may contribute to improved construct stability and potentially reduce mechanical complications, although this requires prospective validation including functional outcome assessment. The present study was primarily designed to evaluate radiographic reproducibility and technical reduction quality rather than comparative clinical effectiveness.

In this context, our findings align closely with the theoretical framework proposed by Minkus and Scheibel,^15^ who demonstrated that a structured stepwise reduction protocol—with controlled fragment handling, temporary transhumeral K-wire retention, and targeted medial calcar reconstruction—facilitates reproducible anatomic restoration. Kwisda et al^12^ subsequently reported excellent functional outcomes and, notably, an absence of primary surgical-technique–related complications when using this standardized method, suggesting that improved intraoperative control may translate into greater initial construct stability. However, their study lacked a comparator group, limiting conclusions about relative effectiveness. By directly contrasting the standardized protocol with conventional, nonstandardized fixation, our results build on this earlier work and provide comparative evidence that the standardized protocol is associated with improved reduction quality compared with conventional nonstandardized approaches.

Although no significant difference in overall NSA change was observed between the standardized and conventional techniques, both techniques were associated with comparable restoration of head-shaft alignment. The smaller post-operative NSA variation in Group 1 (SD 6.3° vs. 13.7°), however, may indicate more consistent control of the reduction. This finding is supported by previous work showing that structured reduction steps and temporary transhumeral K-wire stabilization help maintain alignment during plate fixation.^11^^,^^27^

Medial support remains a key determinant of stability and outcomes in shoulder-preserving treatment of proximal humeral fractures, particularly in elderly patients.^26^ Several augmentation strategies may improve stability but are associated with potential complications.^5^^,^^18^^,^^24^ In our cohort, medial support was achieved more frequently with the standardized reduction and retention technique than with conventional fixation. This is consistent with evidence that nonanatomic reduction increases the risk of secondary displacement,^29^ whereas anatomic reduction is associated with lower complication rates and better clinical outcomes,^25^ highlighting the importance of precise initial reduction.

### Limitations

This study has several limitations that should be taken into account when interpreting the results. First, its retrospective design carries an inherent risk of selection bias and unmeasured confounding, as treatment allocation was surgeon-dependent despite exact fracture type matching and regression adjustment for age. Learning curve effects and surgeon-level confounding therefore cannot be fully excluded. Second, detailed functional outcomes and long-term follow-up were not assessed. Although mean follow-up duration and complication rates requiring revision surgery are reported, patient-reported outcome measures are being evaluated in a dedicated prospective study currently in preparation. The observed difference in revision surgery rates should be interpreted cautiously; the study was not powered to detect differences in complication outcomes, follow-up duration differed numerically between groups, and retrospective selection bias cannot be excluded. Third, reduction quality was assessed using post-operative imaging (radiographs and, in some cases, CT scans), which may be influenced by imaging modality, acquisition technique, and timing, particularly for neck–shaft angle measurements in varus-displaced fractures. As post-operative imaging was obtained between the day of surgery and six weeks post-operatively, some measurement variability cannot be excluded. Fourth, the conventional group was intentionally heterogeneous and reflects the variability of real-world nonstandardized surgical practice, which strengthens clinical applicability but limits strict comparability. While the SRS is an objective and validated assessment tool, radiographic interpretation remains partly observer-dependent. Finally, because this study was conducted at a single center by surgeons with substantial experience in the standardized technique, the generalizability of the results to institutions or surgeons with varying expertise may be limited.

## Conclusion

The standardized reduction and retention technique was associated with more reliable anatomic reduction and medial calcar reconstruction than conventional methods, leading to more consistent post-operative alignment, which may reflect improved construct stability. These findings suggest that structured, stepwise protocols may enhance surgical reproducibility and the quality of fracture reduction. Prospective studies are needed to determine whether these radiographic advantages translate into measurable improvements in function and lower complication rates.

## Disclaimers:

Funding: No funding was disclosed by the authors.

Conflicts of interest: Markus Scheibel is a consultant for Advita Ortho and Stryker Orthopedics. Any additional authors, their immediate families, and any research foundations with which they are affiliated have not received any financial payments or other benefits from any commercial entity related to the subject of this article.

## Data Transparency

The authors affirm that all data supporting the findings of this study are available upon reasonable request.
